# A Progressive dPDOA Localization Method with Quality Control

**DOI:** 10.3390/s26217067

**Published:** 2026-11-09

**Authors:** Quanyue Li, Min Zhang, Guiling Wu, Chao Bo, Shaoming Wang

**Affiliations:** 1 School of Integrated Circuits, Shanghai Jiao Tong University, Shanghai 200240, China; 2 Nanjing Electronic Equipment Research Institute, Nanjing 211100, China; 3 College of Electronic Science and Technology, National University of Defense Technology, Changsha 410073, China

**Keywords:** differential phase difference of arrival (dPDOA), positioning success rate, quality control, standard deviation of range across multiple heights (stdR)

## Abstract

**Highlights:**

**What are the main findings?**

- A progressive dPDOA localization chain integrates TDOA direction finding, multi-height grid search, LAMBDA integer ambiguity fixing, and nonlinear least-squares fine positioning, enabling reliable high-precision positioning without prior information.
- A quality-control mechanism based on multi-height range standard deviation (stdR) effectively eliminates false samples caused by multi-peak cost functions using only coarse search results.

**What is the implication of the main finding?**

- The proposed stdR quality control significantly improves localization success rates (from 87.04% to 94.07% at 10° RMS phase noise; from 48.10% to 68.11% at 30° RMS) without additional computational cost.
- The progressive chain structure provides clear input–output relationships at each step, offering intervention points for quality control and ensuring algorithmic convergence.

**Abstract:**

To address unreliable positioning caused by phase ambiguity and noise in phase difference of arrival (PDOA) systems, this paper proposes a progressive differential phase difference of arrival (dPDOA) localization algorithm with multi-height consistency-based quality control. The algorithm uses dPDOA as the observation model. First, TDOA direction finding obtains a target azimuth prior, reducing the 3D search to a ray. Then, a coarse range search under multiple height hypotheses gives a rough position. The LAMBDA method then fixes integer ambiguities, and a nonlinear least-squares iteration yields the final high-precision result. For reliability, a quality-control step based on the standard deviation of range across multiple heights (stdR) is applied after coarse positioning. By exploiting the closeness of range estimates under different height hypotheses, stdR rejects spurious samples caused by cost-function multimodality. Its computation is simple, using only intermediate coarse-search results, and enables efficient screening of reliable samples before the complex ambiguity-fixing stage. A localization is considered successful when the 3D positioning error is below 8% of the true range. Compared with the same progressive chain without quality control, under 30° RMS single-difference phase noise, the stdR quality control increases the positioning success rate from 48.10% to 68.11%, reduces the median localization error from 38.06 km to 13.02 km, and retains 60.03% of the successful samples.

## 1. Introduction

Passive source localization techniques estimate the target position using electromagnetic signals radiated by the target itself or third-party signals reflected by the target, without actively transmitting electromagnetic waves. They offer significant advantages, including strong concealment, strong anti-interference capability, and flexible deployment, and have substantial application value in electronic reconnaissance, early warning and surveillance, electromagnetic spectrum sensing, and unmanned aerial vehicle (UAV) swarm cooperation [1,2]. Based on the type of observation information, passive source localization methods mainly include schemes using angle of arrival (AOA), time of arrival (TOA), time difference of arrival (TDOA), frequency difference of arrival (FDOA), and phase difference of arrival (PDOA) [3,4]. The PDOA localization scheme achieves high-precision target positioning by exploiting carrier phase differences collected by multiple receiving stations [5]. Compared with TDOA, PDOA offers higher ranging sensitivity and can achieve high accuracy under short-baseline or single-pulse conditions [6] and thus shows broad application prospects in electronic reconnaissance, passive surveillance, and UAV swarm cooperative localization. However, high sensitivity is accompanied by high ambiguity [7]: for civil aviation airborne weather radar signals in the 9–10 GHz band, when the receiving station spacing is on the order of hundreds of meters, the integer ambiguity of differential phase observations can reach tens of thousands of cycles. Conventional ambiguity resolution methods typically rely on prior information such as initial position or motion [8] and often fail in passive scenarios without external guidance, leading to large jumps or even entirely erroneous positioning results. Moreover, factors such as signal quality can introduce spurious localizations, resulting in a serious false alarm problem. These factors make reliable positioning in fully passive scenarios particularly challenging. Existing research on differential phase difference of arrival (dPDOA) localization has been conducted from multiple perspectives. For joint localization and performance analysis, Ref. [9] derived the CRLB for TDOA/FDOA/dPDOA under both phase-synchronized and phase-asynchronous conditions. For ambiguity resolution, Refs. [10,11] exploited the common integer ambiguity of equal-length baselines and eliminated it by phase subtraction, enabling single-pulse instantaneous ranging. Ref. [12] addressed the nonlinearity caused by 2π phase ambiguity using interferometer-assisted second-order phase differencing with self-iterative correction, and Ref. [13] combined cross-correlation interpolation for coarse time-delay estimation with a second-stage phase ambiguity resolution to obtain high-precision time-delay differences. In terms of station geometry optimization, Ref. [14] established an optimization model with the trace of the CRLB matrix as the objective function and employed a particle swarm optimization algorithm to determine the optimal geometric configuration for dPDOA-based localization. With respect to baseline design, Ref. [15] proposed a novel baseline arrangement scheme that utilized two relatively short, parallel, symmetric real baselines to estimate the target range by combining phase differences to cancel large angle-dependent terms. Ref. [16] mapped two-dimensional array elements onto a one-dimensional virtual baseline through orthogonal projection, forming a large-aperture equivalent array, and derived the target position using range differences obtained from phase-difference measurements, and Ref. [17] validated the accuracy advantage of joint carrier-phase and code-phase observables under short-baseline conditions. For systematic-error calibration, Ref. [18] employed positioning reference units (PRUs) to calibrate dPDOA systematic errors and verified the accuracy through field measurements. Despite these advances, existing methods still face two major challenges under fully passive conditions: efficient integer ambiguity resolution and effective suppression of spurious localization results. These challenges constitute the motivation for the present study.

To address these limitations, this paper proposes a progressive dPDOA localization framework with quality control. The main technical contributions are twofold. (1) A progressive coarse-to-fine localization chain is developed, consisting of TDOA coarse direction finding, azimuth-constrained grid search for coarse positioning, LAMBDA integer ambiguity fixing, and nonlinear least-squares fine positioning. Using the TDOA azimuth prior, the chain compresses the 3D search space into a ray, obtains a reliable coarse position through multi-height hypothesis searches, and provides a high-quality initial point for ambiguity fixing, thereby enabling high-precision positioning without prior information. (2) To mitigate the “false peak” problem caused by cost-function multimodality, an stdR-based pre-ambiguity-fixing quality-control metric is proposed. It exploits the consistency of range estimates across multiple height hypotheses to reject unreliable samples before the complex ambiguity-fixing stage. This metric reuses the intermediate results of the coarse search and has low computational cost and efficiently rejects unreliable samples before the complex ambiguity-fixing stage. Monte Carlo simulations under different phase-noise and TDOA-error levels are conducted to validate the proposed method, and the results show that quality control improves the success rate and reduces the median localization error.

## 2. Progressive High-Precision Localization Chain

**Figure 1:**
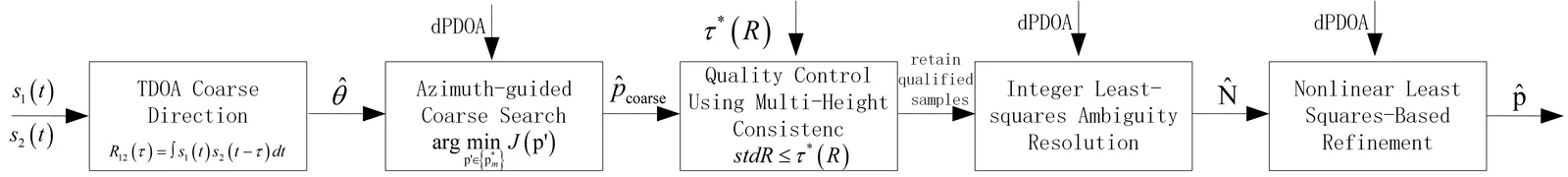
Flowchart of the progressive positioning algorithm.

The proposed progressive dPDOA localization algorithm is illustrated in Figure 1. The algorithm takes multi-station dPDOA observations and TDOA measurements as inputs and outputs the final 3D target position. It consists of five layers.

In Layer 1 (TDOA coarse direction finding), the received signals, *s*_1_(*t*) and *s*_2_(*t*), from the longest baseline are used for cross-correlation time-delay estimation, and the azimuth prior, $\hat{\theta }$, is obtained.

In Layer 2 (azimuth-guided multi-height coarse search), the 3D search is compressed into a ray determined by $\hat{\theta }$, and a one-dimensional range search is performed under multiple height hypotheses. For each height, the best range estimate, $R_{m}^{*}$, and the corresponding minimum cost, $J_{m}^{*}$, are recorded, and the coarse position, ${\hat{p}}_{\mathrm{coarse}}$, is obtained.

In Layer 3 (stdR-based quality control), the stdR metric is computed from the range estimates, RmiRmi, across heights and compared with the threshold, *τ*_stdR_. If stdR ≤ *τ*_stdR_, the sample is considered reliable and proceeds to Layer 4; otherwise, it is rejected as a failure.

In Layer 4 (LAMBDA integer ambiguity fixing), the coarse position, ${\hat{p}}_{\mathrm{coarse}}$, is used as the initial linearization point. The floating ambiguity solution, ${\hat{N}}_{\mathrm{float}}$, and its covariance, $Q_{\hat{N}}$, are computed, and the LAMBDA method is employed to fix the integer ambiguities, $\hat{N}$.

In Layer 5 (nonlinear least-squares refinement), the fixed ambiguities are used to refine the target position by nonlinear least-squares optimization with the trust-region reflective algorithm, yielding the final position estimate, $\hat{p}$.

### 2.1. First Layer: TDOA Coarse Direction Finding

The system uses the longest baseline for cross-correlation time-delay estimation. Let the signals received at two stations be *s*_1_(*t*) and *s*_2_(*t*). The cross-correlation function is defined in Equation (1):

(1) $$R_{12}\left(\tau \right)=\int s_{1}\left(t\right)s_{2}\left(t-\tau \right)\mathrm{dt}.$$

The time-delay estimate, $\hat{\tau }$, is the argument that maximizes the cross-correlation, as given in Equation (2):

(2) $$\hat{\tau }=\arg\max_{\tau }R_{12}\left(\tau \right).$$

The time-difference measurement accuracy, *σ*_Δ_*_t_*, is related to the signal bandwidth, *B*, and signal-to-noise ratio (SNR) by $\sigma_{\Delta t}\propto 1/\left(B\sqrt{\mathrm{SNR}}\right)$ [19]. For typical airborne weather radar pulses, sufficient time-difference accuracy is easy to achieve.

Let the baseline length (distance between the two stations) be *d* and the target azimuth (referenced to the array normal) be *θ*. The relationship between the time difference *τ* and *θ* is approximately given by Equation (3) [8]:

(3) $$\tau =\frac{d}{c}\mathrm{sin\theta},$$

where *c* is the speed of light. The azimuth estimate, $\hat{\theta }$, and its error, *σ_θ_*, are then given by Equation (4):

(4) $$\sigma_{\theta }=\frac{c}{\mathrm{dcos\theta}}\sigma_{\Delta t}.$$

This layer provides an azimuth prior without external information. Using the longest baseline for cross-correlation time-delay estimation, it compresses the 3D search into a ray and avoids reliance on prior position or motion. The computation is simple and suitable for practical processing.

### 2.2. Second Layer: Azimuth-Based Coarse Positioning

**Figure 2:**
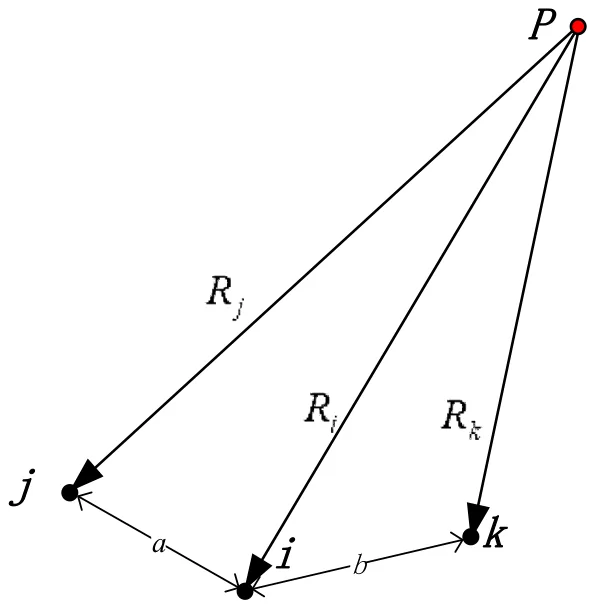
Schematic diagram of the dPDOA observation model.

This paper adopts a centrosymmetric double-difference construction. As shown in Figure 2, point P denotes the signal radiation location; for a fixed set of three receiving stations, *i* (center station), *j* and *k* (symmetric slave stations), the dPDOA observation is defined as Equation (5) [11]:

(5) Δ∇*ϕ_ijk_* = *ϕ_j_* + *ϕ_k_* − 2*ϕ_i_*.

The observation equation is given in Equation (6):

(6) $$\Delta \nabla \phi_{\mathrm{ijk}}=\frac{2\pi }{\lambda }\left(R_{j}+R_{k}-2R_{i}\right)+2\pi N_{\mathrm{ijk}}+\Delta \nabla \beta_{\mathrm{ijk}}+\Delta \nabla \epsilon_{\mathrm{ijk}},$$

where

- *λ* is the signal wavelength (in meters);
- *R_i_* = ||p − r*_i_*|| is the geometric distance from the target to station *i*, with similar definitions for *R_j_*,*R_k_*;
- *N_ijk_* = *N_j_* + *N_k_* − 2*N_i_* ∈ ℤ is the combined integer ambiguity;
- Δ∇*β_ijk_* = *β_j_* + *β_k_* − 2*β_i_* is the combined channel phase deviation (systematic bias after calibration);
- Δ∇*ϵ_ijk_* = *ϵ_j_* + *ϵ_k_* − 2*ϵ_i_* is the combined noise, following $N(0,6\sigma_{\phi }^{2}).$

The per-station phase noise is assumed to be independent with equal variance, $\sigma_{\phi }^{2}$. For a single dPDOA observation, the combined noise is Δ∇*ϵ_ijk_* = *ϵ_j_* + *ϵ_k_* − 2*ϵ_i_*, so its variance is $6\sigma_{\phi }^{2}$. Since different dPDOA observations share the same center station, their combined noises are correlated through the common term 2*ϵ_i_*. For two observations sharing only the center station, the covariance is $4\sigma_{\phi }^{2}$, and the correlation coefficient is 2/3. This correlation is retained in the subsequent weighted estimation.

This construction takes the central reference station as the symmetry center. When the station spacings *a* = *b*, most of the geometric distance terms cancel out under symmetry, directly providing ambiguity information. Let 𝒮 be the set of dPDOA observations used by the system, each corresponding to a triplet, (*i*,*j*,*k*) ∈ 𝒮, i.e., a center station, *i*, and two symmetric slave stations, *j*,*k*. For any candidate position, *p*’, the theoretical phase difference (without ambiguity) for each dPDOA observation is first computed as Equation (7):

(7) $$\Delta {\tilde{\phi }}_{\mathrm{ijk}}\left(p^{\prime }\right)=\frac{2\pi }{\lambda }\left(∥p^{\prime }-r_{j}∥+∥p^{\prime }-r_{k}∥-2∥p^{\prime }-r_{i}∥\right),$$

where r*_i_*, r*_j_*, r*_k_* are the nominal coordinates of the receiving stations (including site errors).

For the measured dPDOA phase difference, $\Delta \nabla \phi_{\mathrm{ijk}}^{\mathrm{meas}}$, the difference from the theoretical value mainly comes from geometric deviation and integer ambiguity. The ambiguity estimate is defined as Equation (8):

(8) $${\hat{N}}_{\mathrm{ijk}}\left(p^{\prime }\right)=\mathrm{round}\left(\frac{\Delta \nabla \phi_{\mathrm{ijk}}^{\mathrm{meas}}-\Delta {\tilde{\phi }}_{\mathrm{ijk}}\left(p^{\prime }\right)}{2\pi }\right),$$

where round(·) denotes rounding to the nearest integer with ties away from zero. This estimate represents the integer ambiguity that makes the measured value closest to the theoretical value at the candidate position, p’.

The phase residual for this observation at p’ is then given by Equation (9):

(9) $$r_{\mathrm{ijk}}\left(p^{\prime }\right)=∥\Delta \nabla \phi_{\mathrm{ijk}}^{\mathrm{meas}}-\Delta {\tilde{\phi }}_{\mathrm{ijk}}\left(p^{\prime }\right)-2\pi {\hat{N}}_{\mathrm{ijk}}\left(p^{\prime }\right)∥.$$

Equations (6)–(9) are derived from the dPDOA observation model given in Equation (5).

Summing the squared residuals of all dPDOA observations in the system gives the spatial coherence cost function in Equation (10):

(10) $$J\left(\mathrm{p’}\right)=\sum_{\left(i,j,k\right)\in 𝒮}r_{\mathrm{ijk}}^{2}\left(\mathrm{p’}\right).$$

This cost function does not rely on any ambiguity prior; it uses only the phase-difference observations themselves and the current station geometry. When the candidate position, p’, is close to the true target position, the residuals for all baselines are near the phase-noise level, and *J*(p’) attains a minimum.

**Figure 3:**
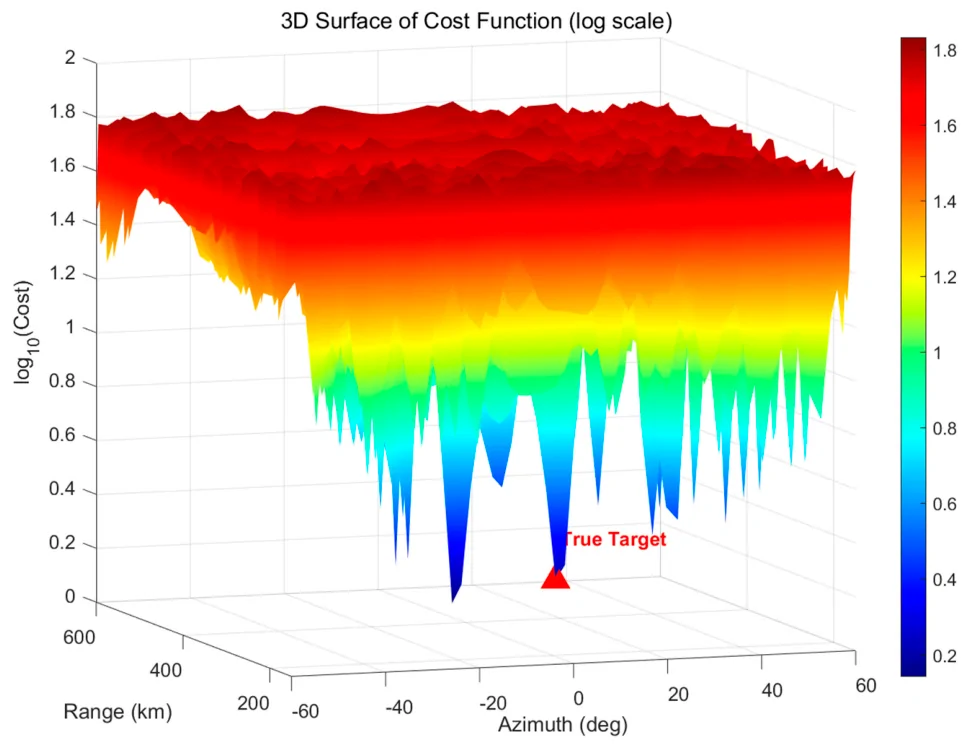
Illustration of the cost function.

Figure 3 shows the cost function over the observation region for a target at 10° azimuth, a range of 300 km, and a height of 8 km; after calibration, the site error RMS is 1 mm, the channel error RMS is 1.5°, the single-difference phase measurement noise RMS is 10° (i.e., $\sigma_{\phi_{\mathrm{single}}}=10^{\circ }$), the original phase noise (per receiving station) RMS is $\sigma_{\phi_{\mathrm{orig}}}=\sigma_{\phi_{\sin\mathrm{gle}}}/\sqrt{2}\approx 7.07^{\circ }$, and $\sigma_{\mathrm{dPDOA}}=\sqrt{6}\times 7.07^{\circ }\approx 17.3^{\circ }$. Station coordinates and baseline pairs are detailed in Section 3.

Using the azimuth prior, ${\hat{\theta }}_{\mathrm{TDOA}}$, obtained in Section 2.1, the search space is restricted to the ray determined by that direction. To handle the unknown target height, three typical heights are chosen: *h*_1_ = 3000 m, *h*_2_ = 5000 m, and *h*_3_ = 8000 m. For each height, a one-dimensional range search is performed. The range search covers the possible far-near distances (e.g., 0–600 km) with a step size, Δ*R*.

For each height hypothesis, *h_m_*, candidate points are generated as $\mathrm{p’}={\left[R\sin{\hat{\theta }}_{\mathrm{TDOA}},\;R\cos{\hat{\theta }}_{\mathrm{TDOA}},\;h_{m}\right]}^{T}$, where *R* traverses the range grid. The cost, *J*(p’), is computed for each candidate, and the best range, $R_{m}^{*}=\mathrm{argmin}J$, and the corresponding minimum cost, $J_{m}^{*}$, are recorded. Finally, the result with the smallest cost among the three heights is taken as the output of the second-step coarse positioning in Equation (11):

(11) $${\hat{p}}_{\mathrm{coarse}}=\arg\min_{\mathrm{p’}\in \{p_{m}^{*}\}}J\left(\mathrm{p’}\right).$$

Equation (10) defines the spatial coherence cost function used in this work, and Equation (11) gives the corresponding coarse positioning output. The output, ${\hat{p}}_{\mathrm{coarse}}$, although limited in accuracy (depending on the step size and height hypotheses), is already sufficiently close to the true value to provide the initial linearization point for the subsequent LAMBDA method.

This layer handles coarse positioning under an unknown target height. The centrosymmetric double-difference construction defines a cost function independent of any ambiguity prior, and the multi-height hypothesis searches reduce the problem to a set of one-dimensional range searches. The resulting coarse position is accurate enough to serve as a reliable initial linearization point for LAMBDA.

### 2.3. Third Layer: Quality Control Based on Multi-Height Consistency

The proposed multi-height range standard deviation (stdR) quality-control method is based on the idea of consistency checking. The core logic is that the true target position should satisfy multiple independent geometric constraints simultaneously, and the estimates given by different constraints should be consistent with each other.

In the second-step coarse positioning of Section 2.2, the algorithm performs a one-dimensional range search under three typical height hypotheses (*h*_1_ = 3000 m, *h*_2_ = 5000 m, and *h*_3_ = 8000 m), obtaining the best range estimate, $R_{m}^{*}$, for each height. If the candidate position is close to the true target, these range estimates should be roughly the same; conversely, if a false minimum exists at a certain height, the estimated range at that height will deviate significantly from the others. This property can be used to construct a quality-control metric based on multi-height consistency to reject obviously unreliable samples.

The standard deviation of range estimates across heights (stdR) is defined as Equation (12):

(12) $$\mathrm{stdR}=\sqrt{\frac{1}{K}\sum_{m=1}^{K}{\left(R_{m}^{*}-\bar{R}\right)}^{2}},$$

where *K* is the number of height hypotheses used. Equation (12) is the definition of the proposed stdR metric and is original to this work. This metric quantifies the dispersion of the range estimates across different heights. Its mathematical foundation is the consistency check idea: the true target position should satisfy multiple independent geometric constraints, and the estimates from different constraints should be consistent.

For a fixed azimuth, *θ*, the candidate position can be written as p’ = (*R*sin*θ*, *R*cos*θ*, *h*)*^T^*. The geometric distance function is continuously differentiable with respect to *R* and relatively insensitive to changes in *h*. Therefore, near the true position, p*, the cost functions, *J*(p’), under different height hypotheses should attain minima in the same neighborhood of *R*, implying $R_{m}^{*}\approx R$ for all *m*.

When a false minimum appears at a certain height, *h_m_*, the corresponding best range $R_{m}^{*}$ will significantly deviate from the true range, while the estimates at other heights remain close to *R*, causing stdR, defined in Equation (12), to increase. Thus, stdR can be viewed as an outlier detection statistic: if its value is less than a preset threshold, τ*(*R*), the second-step result is considered reliable, and the sample proceeds to subsequent processing; otherwise, it is directly judged as a failure and rejected.

This method is mathematically equivalent to over-determined geometric constraints—the true position must satisfy observation equations at multiple different heights, and the degree of consistency among these equations directly reflects the reliability of the localization. By treating each height as an independent “range finder,” if their readings agree, the target position is credible; otherwise, interference from false minima is suspected. The stdR threshold detection fully exploits the intermediate results of the second-step search without extra computation, providing an efficient pre-quality-control defense line for the progressive localization chain.

The stdR metric provides a pre-ambiguity-fixing reliability check. A real target gives consistent range estimates across heights, while a false peak causes divergence. By comparing stdR with a threshold, unreliable samples are rejected before ambiguity fixing. This check fully reuses the coarse-search results and adds no extra computation.

### 2.4. Fourth Layer: Integer Least-Squares Ambiguity Fixing

After obtaining the coarse positioning result, the system must precisely determine the integer ambiguities for each baseline; this is the key to achieving high-precision positioning. The coarse position, ${\hat{p}}_{\mathrm{coarse}}$, provides a reliable initial point for linearization, making the floating ambiguity solution close to the integer truth. This section uses the LAMBDA method to fix the combined ambiguities, *N_ijk_*.

Linearizing Equation (6) around the initial estimate, $p_{0}={\hat{p}}_{\mathrm{coarse}}$, yields Equation (13) [20]:

(13) z = g(p_0_) − G(p − p_0_) + 2*π*N + ϵ,

where

- z is the vector of multiple dPDOA observations;
- $g\left(p\right)=\frac{2\pi }{\lambda }\left(∥p-r_{j}∥+∥p-r_{k}∥-2∥p-r_{i}∥\right)$ is the geometric function vector;
- $G={\frac{\partial g}{\partial p}|}_{p=p_{0}}$ is the Jacobian matrix;
- N is the corresponding integer ambiguity vector;
- ϵ is the combined noise vector with a covariance matrix, Q*_ϵ_*.

The covariance matrix, Q*_ϵ_*, in Equation (13) is not diagonal. It accounts for the correlation caused by the shared center station and is used in the weighted least-squares estimation of the floating ambiguities, in the subsequent LAMBDA search, and in the final nonlinear least-squares refinement.

The floating ambiguity solution is defined as Equation (14):

(14) $${\hat{N}}_{\mathrm{float}}=\frac{1}{2\pi }\left(z-g\left(p_{0}\right)\right),p_{0}={\hat{p}}_{\mathrm{coarse}}.$$

Its covariance matrix is given by Equation (15):

(15) $$Q_{\hat{N}}=\frac{1}{4\pi^{2}}Q_{\epsilon }^{-1}.$$

The integer least-squares problem in Equation (16) [21] is to find the integer vector that minimizes the weighted squared residual:

(16) N^=arg minN∈ZKN^float−NTQN^−1N^float−N.

This problem is solved by integer decorrelation (Z-transformation) and tree search. Using the coarse position as the initial linearization point, this layer resolves the integer ambiguities. The close initial point makes the floating ambiguity solution near the integer truth and improves the fixing success rate. The LAMBDA method efficiently solves the integer least-squares problem, and the covariance matrix retains the noise correlation caused by the shared center station.

### 2.5. Fifth Layer: Nonlinear Least-Squares Fine Positioning

After the integer ambiguities are successfully fixed in the fourth step, the dPDOA observations become ambiguity-free geometric constraints. The target position can then be further refined by solving a nonlinear least-squares problem. Let the residual vector for the *M*-retained baselines be r(p) ∈ R*^M^*, whose *m*-th component is given by Equation (17):

(17) $$r_{m}\left(p\right)=\frac{1}{\lambda }\left(∥p-r_{j_{m}}∥+∥p-r_{k_{m}}∥-2∥p-r_{i_{m}}∥\right)-N_{i_{m}j_{m}k_{m}}-\frac{\phi_{i_{m}j_{m}k_{m}}}{2\pi },\;m=1,\ldots ,M,$$

where p ∈ R^3^ is the unknown target position, $r_{i_{m}},\;r_{j_{m}},\;r_{k_{m}}$ are the coordinates of the corresponding receiving stations, $N_{i_{m}j_{m}k_{m}}$ is the combined ambiguity fixed in the fourth step, and $\phi_{i_{m}j_{m}k_{m}}$ is the wrapped dPDOA measurement (in radians). The goal is to minimize the sum of squared residuals, as in Equation (18):

(18) $$\hat{p}=\arg\min_{p}F\left(p\right),\;F\left(p\right)=\frac{1}{2}∥r\left(p\right){∥}^{2}=\frac{1}{2}\sum_{m=1}^{M}r_{m}^{2}\left(p\right).$$

Because the geometric term is strongly nonlinear in p, an iterative optimization method is required. This paper employs the trust-region reflective algorithm, which performs well for strongly nonlinear problems; in tests it converged faster and better than the Levenberg–Marquardt algorithm [22] and does not require manual adjustment of the damping factor.

The core idea of the trust-region method is to define a trust-region radius, Δ*_k_*, around the current iterate, p*_k_*, and approximate the original objective function by a quadratic model within that region. Denote the residual vector, r*_k_* = r(p*_k_*), and the Jacobian matrix, $J_{k}={\frac{\partial r}{\partial p}|}_{p_{k}}\in R^{M\times 3}$. The second-order Taylor expansion approximation of the objective function is given by Equation (19):

(19) $$q_{k}\left(s\right)=\frac{1}{2}∥r_{k}+J_{k}s{∥}^{2}=\frac{1}{2}∥r_{k}{∥}^{2}+s^{T}J_{k}^{T}r_{k}+\frac{1}{2}s^{T}J_{k}^{T}J_{k}s.$$

The algorithm seeks a trial step, s*_k_*, within the trust region, ∥s∥≤Δ*_k_*, that minimizes *q_k_*(s). To combine global convergence and local superlinear convergence, the trust-region reflective algorithm restricts the search direction to a two-dimensional subspace spanned by the gradient direction, $g_{k}=J_{k}^{T}r_{k}$, and an approximate Newton direction (obtained by solving $J_{k}^{T}J_{k}s=-g_{k}$ with a preconditioned conjugate gradient method). This strategy effectively combines the global convergence property of the steepest descent method and the fast local convergence of Newton’s method.

After obtaining the trial step, s*_k_*, the ratio of the actual reduction to the predicted reduction is computed as Equation (20) [22]:

(20) $$\rho_{k}=\frac{{\mathrm{ared}}_{k}}{{\mathrm{pred}}_{k}}=\frac{F\left(p_{k}\right)-F\left(p_{k}+s_{k}\right)}{q_{k}\left(0\right)-q_{k}\left(s_{k}\right)}.$$

The trust-region radius, Δ*_k_*, is adjusted according to *ρ_k_* defined in Equation (20):

- If *ρ_k_* > 0.75, the model is accurate, and the trust region is expanded to accelerate convergence: Δ*_k_*_+1_ = min(2Δ*_k_*, Δ_max_);
- If *ρ_k_* < 0.25, the model is distorted, and the trust region is shrunk to ensure robustness: Δ*_k_*_+1_ = 0.5Δ*_k_*;
- If *ρ_k_* ≤ 0, the trial step is rejected, and the trust region is reduced directly without updating the point.

When *ρ_k_* > 0, the trial step is accepted, and p*_k_*_+1_ = p*_k_* + s*_k_*; otherwise, the position remains unchanged. The iteration continues until convergence (e.g., step size ∥s*_k_*∥<10^−4^ m or a maximum of 30 iterations).

Once the ambiguities are fixed, the dPDOA observations become ambiguity-free geometric constraints, and the positioning problem reduces to a nonlinear least-squares problem. The trust-region reflective algorithm handles the strong nonlinearity well, converges faster and more robustly than the Levenberg–Marquardt algorithm, and requires no manual damping adjustment. This layer yields the final high-precision position estimate.

## 3. Simulation Verification and Performance Comparison

This section validates the proposed progressive phase-difference positioning algorithm through Monte Carlo simulations, focusing on the improvement in localization results brought by quality control.

### 3.1. Simulation Environment and Parameters

The simulation scenario is wide-area airborne target surveillance. Target heights are uniformly random between 0 and 10,000 m, horizontal ranges cover 150–600 km, and azimuths cover the main sector of ±62.5°. The receiving station coordinates are listed in Table 1, comprising 8 horizontal baseline pairs and 8 mixed baseline pairs, as shown in Table 2. The main error source parameters are given in Table 3.

The success threshold is defined as a positioning error below 8% of the true range, which ensures reliable track initiation in downstream filtering. Reference [23] shows that track initialization errors above 50 km cause the Kalman filter to diverge; the 8% criterion keeps the absolute error well below this boundary across the entire observation range. The target observation range is restricted to above 150 km, and all subsequent quality-control results and performance analyses are reported only for R ≥ 150 km. In the simulations, the measurement noise is generated using the full covariance matrix, Q*_ϵ_*, defined in Section 2.2, including the off-diagonal terms due to the shared center station.

Unless otherwise stated, 200 independent Monte Carlo trials are performed for each range–azimuth grid point under each phase-noise level.

### 3.2. Statistical Calibration of the stdR Threshold

The stdR quality control introduced in Section 2.3 requires a threshold, *τ*_stdR_, to decide whether a coarse-positioning result is reliable. The choice of this threshold is not arbitrary; it is derived from a statistical analysis of Monte Carlo data. This section describes the calibration procedure.

Quality-control metrics: Two complementary metrics are used to evaluate a candidate threshold, *τ*:

Post-acceptance success rate, *P_S_*_|_*_A_* (*τ*,*R*), is defined in Equation (21): among the samples that pass quality control at range R, for the fractions that are truly successful,

(21) $$P_{S|A}(\tau ,R)=\frac{N_{\mathrm{acc},\mathrm{succ}}(\tau ,R)}{N_{\mathrm{acc}}(\tau ,R)}.$$

where *N_acc_*(*τ*,*R*) is the number of samples that pass quality control at range *R*, and *N_acc_*_,_*_succ_*(*τ*,*R*) is the number of accepted samples that are truly successful.

Success-sample retention rate, *P_D_* (*τ*,*R*), is defined in Equation (22): among all successful samples at range R, for the fraction retained by quality control,

(22) $$P_{D}(\tau ,R)=\frac{N_{\mathrm{acc},\mathrm{succ}}(\tau ,R)}{N_{\mathrm{succ}}(R)}.$$

where *N_succ_*(*R*) is the total number of successful samples at range R. *P_D_* (*τ*,*R*) measures how many successful samples are retained by quality control.

A successful localization is defined as one whose 3D positioning error is less than 8% of the true range (see Section 3.1).

Calibration criterion: A good threshold keeps both metrics high. Since *P_S_*_|_*_A_* and *P_D_* respond to *τ* in opposite directions, the threshold is chosen as the maximin compromise in Equation (23):

(23) τ*R=argmaxτ min{PS|Aτ,R,PD(τ,R)}.

**Figure 4:**
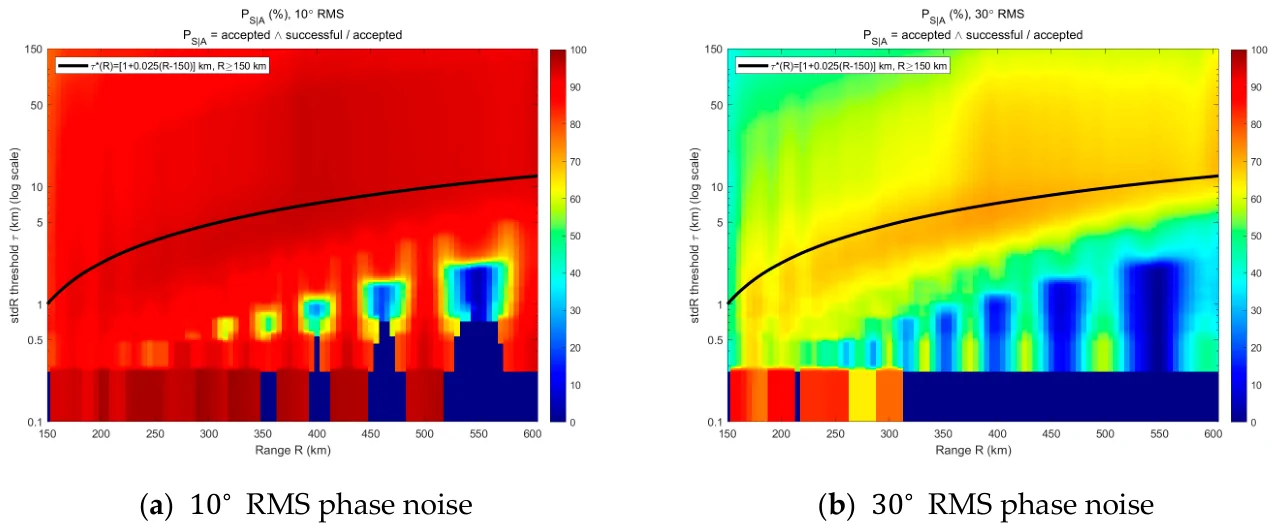
Color-coded *P_S_*_|_*_A_* versus range and stdR threshold.

**Figure 5:**
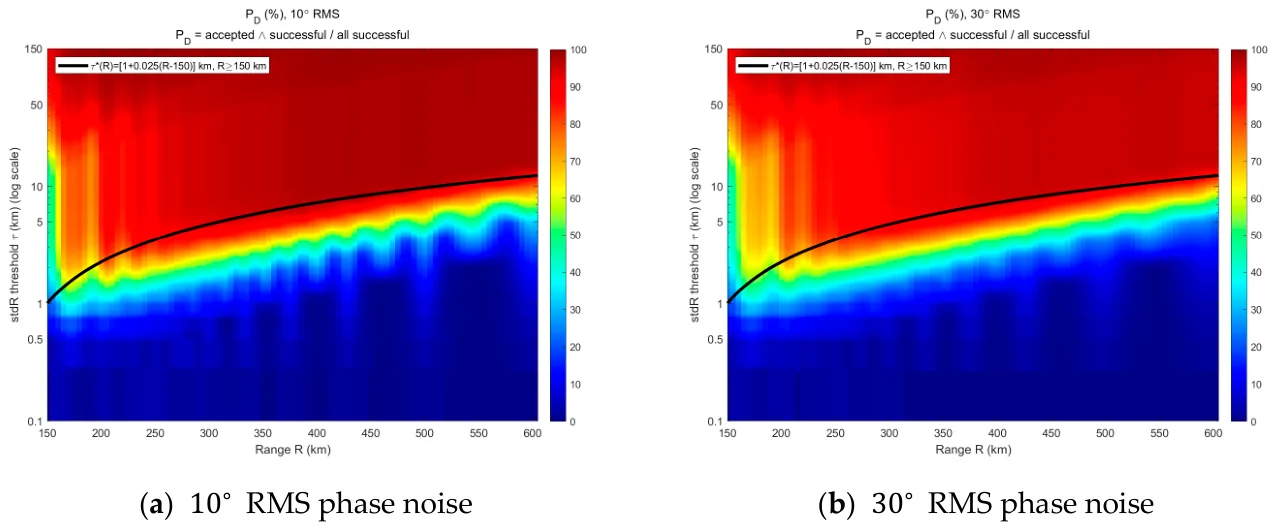
Color-coded *P_D_* versus range and stdR threshold.

Figure 4 and Figure 5 present the two quality-control metrics as color-coded functions of range, R, and stdR threshold, τ. Figure 4a and Figure 4b give *P_S_*_|_*_A_* under 10° and 30° RMS phase noise, respectively. Figure 5a and Figure 5b give *P_D_* under 10° and 30° RMS phase noise, respectively. Here *P_S_*_|_*_A_* is the post-acceptance success rate, i.e., among the samples that pass quality control, this is the fraction that is truly successful; *P_D_* is the success-sample retention rate, i.e., among all successful samples, this is the fraction retained by quality control. In each subplot, the horizontal axis is range, R (km), the vertical axis is the stdR threshold, τ (km, log scale), and the colorbar denotes the corresponding percentage (%). The black curve in all four subplots is the fitted threshold locus, τ*(*R*), obtained from the maximin criterion in Equation (23) and expressed explicitly by Equation (24). A localization is considered successful if the 3D positioning error is less than 8% of the true range, and only samples with R ≥ 150 km are counted.

Two observations follow. At the shortest ranges within the observation window, for each noise level, the bright regions of the *P_S_*_|_*_A_* and *P_D_* heatmaps overlap only at a very small *τ*: increasing τ in this range admits many failed samples, so *P_S_*_|_*_A_* drops faster than *P_D_* rises. As range increases, the bright regions shift to a larger *τ*, because the stdR distributions of successful and failed samples separate progressively with range. The locus of τ*(*R*), defined in Equation (23), traces an approximately linear path in the (*R*,*τ*) plane at both noise levels.

Threshold expression: Fitting the locus of τ*(*R*) gives Equation (24):

(24) τ*(*R*) = [1 + 0.025(*R* − 150)]km, *R* ≥ 150 km.

Since the present study only considers R ≥ 150 km, Equation (24) is applied only over this range, where τ and R are in km. The black curve in Figure 4 and Figure 5 shows that Equation (24) passes through the common bright band of both metrics at both noise levels over the considered range, confirming that the two-parameter form captures the essential structure of the optimum.

The robustness of Equation (24) was assessed by varying the slope by ±20% (0.020–0.030) and the breakpoint 150 km by ±25 km. Over the 150–600 km range the resulting *P_S_*_|_*_A_* and *P_D_* changed by less than 3 percentage points, indicating that the threshold is a robust pre-quality-control gate rather than a finely tuned parameter.

### 3.3. Quality-Control Effectiveness Analysis

Following the simulation setup in Section 3.1, 200 Monte Carlo trials are performed for each range–azimuth grid point under each phase-noise level (10° and 30° RMS).

**Figure 6:**
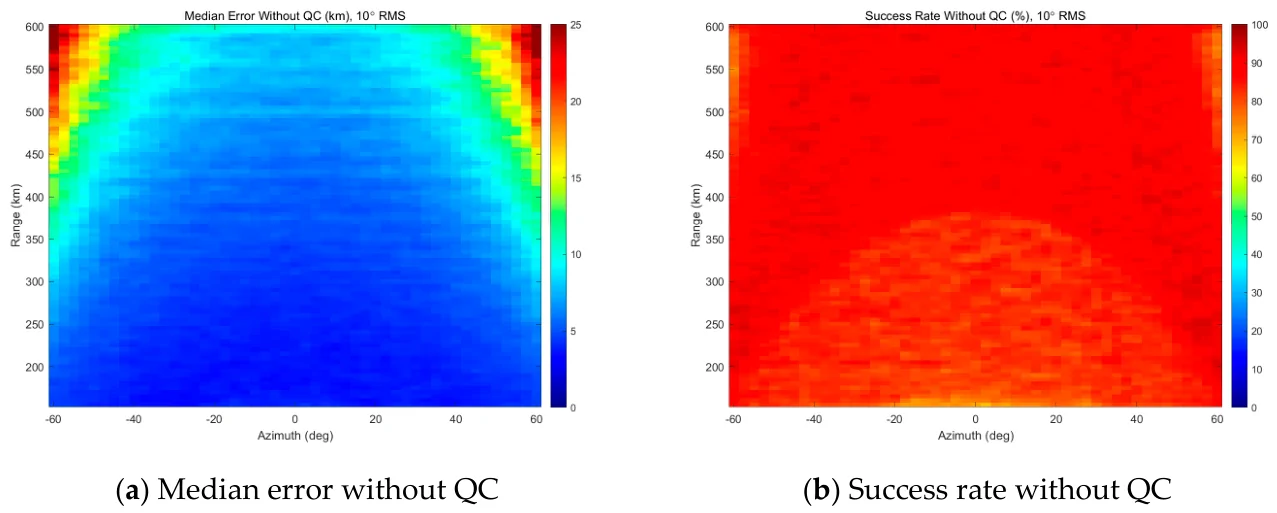
Localization error distribution and success rate without quality control (single-difference phase noise of 10° RMS).

**Figure 7:**
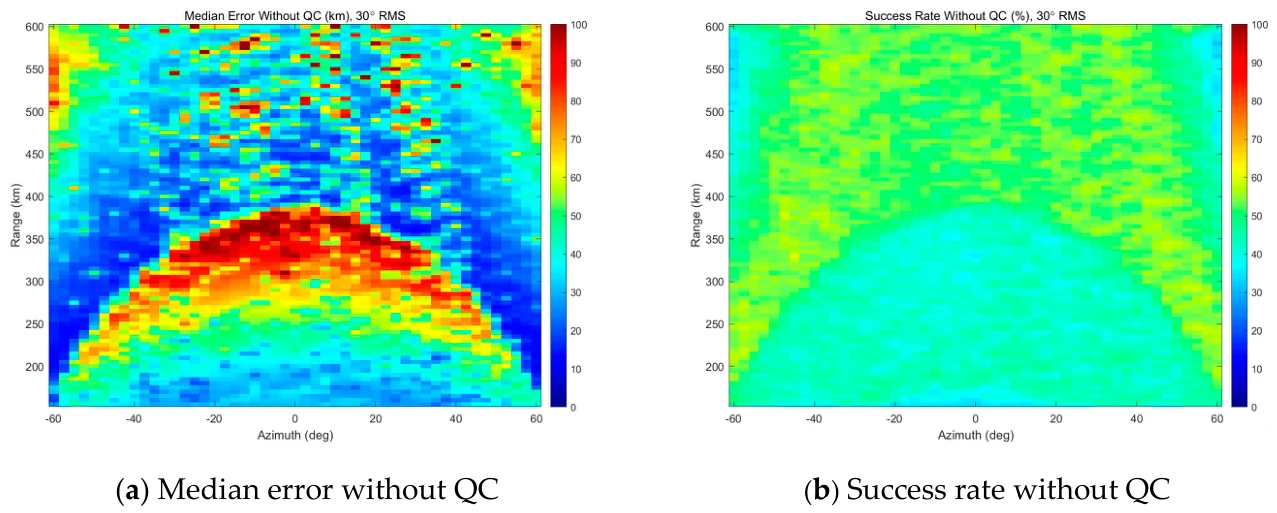
Localization error distribution and success rate without quality control (single-difference phase noise of 10° RMS).

Figure 6 and Figure 7 show the localization performance without quality control over the observation range of R ≥ 150 km. Figure 6a and Figure 6b present the median localization error distribution and the success rate under 10° RMS phase-difference measurement noise, respectively. Figure 7a,b present the corresponding median localization error distribution and success rate under 30° RMS phase-difference measurement noise. In Figure 6a and Figure 7a, the horizontal axis is azimuth (deg), the vertical axis is range (km), and the colorbar denotes the median localization error (km). In Figure 6b and Figure 7b, the horizontal axis is azimuth (deg), the vertical axis is range (km), and the colorbar denotes the success rate (%). A localization is considered successful when the 3D positioning error is less than 8% of the true range.

**Figure 8:**
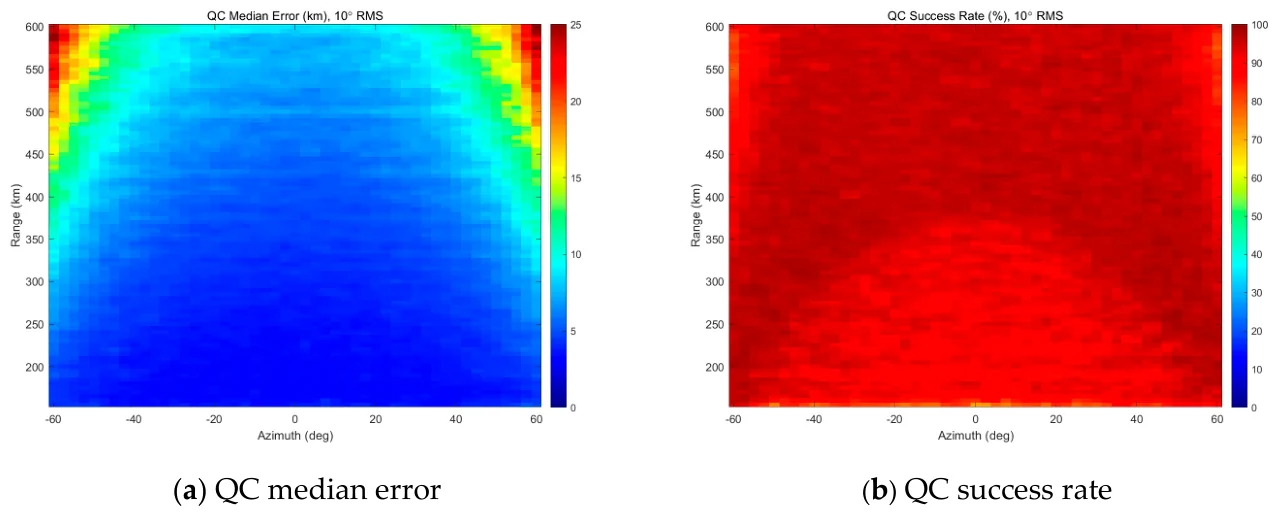
Localization error distribution and success rate with quality control (single-difference phase noise of 10° RMS).

**Figure 9:**
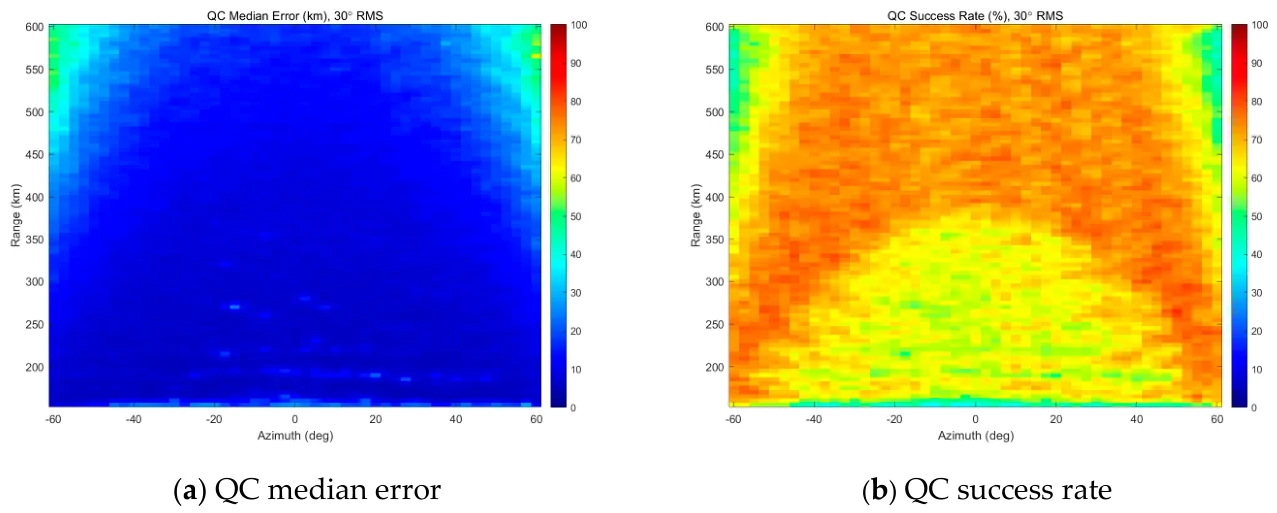
Localization error distribution and success rate with quality control (single-difference phase noise of 30° RMS).

The stdR quality-control threshold follows Equation (24). Figure 8 and Figure 9 present the localization performance with the stdR quality control applied over the observation range of R ≥ 150 km. Figure 8a and Figure 8b show the median localization error distribution and the success rate under 10° RMS phase noise, respectively. Figure 9a,b show the corresponding median localization error distribution and success rate under 30° RMS phase noise. In Figure 8a and Figure 9a, the horizontal axis is azimuth (deg), the vertical axis is range (km), and the colorbar denotes the median localization error (km). In Figure 8b and Figure 9b, the horizontal axis is azimuth (deg), the vertical axis is range (km), and the colorbar denotes the success rate (%). A localization is considered successful when the 3D positioning error is less than 8% of the true range.

**Figure 10:**
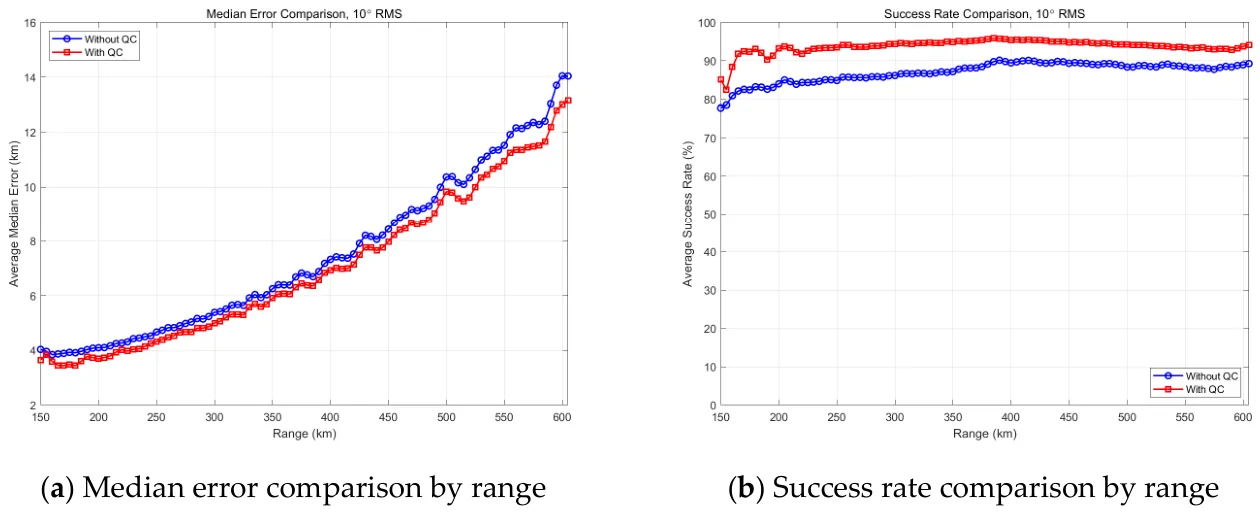
Comparison with and without quality control (single-difference phase noise of 10° RMS).

**Figure 11:**
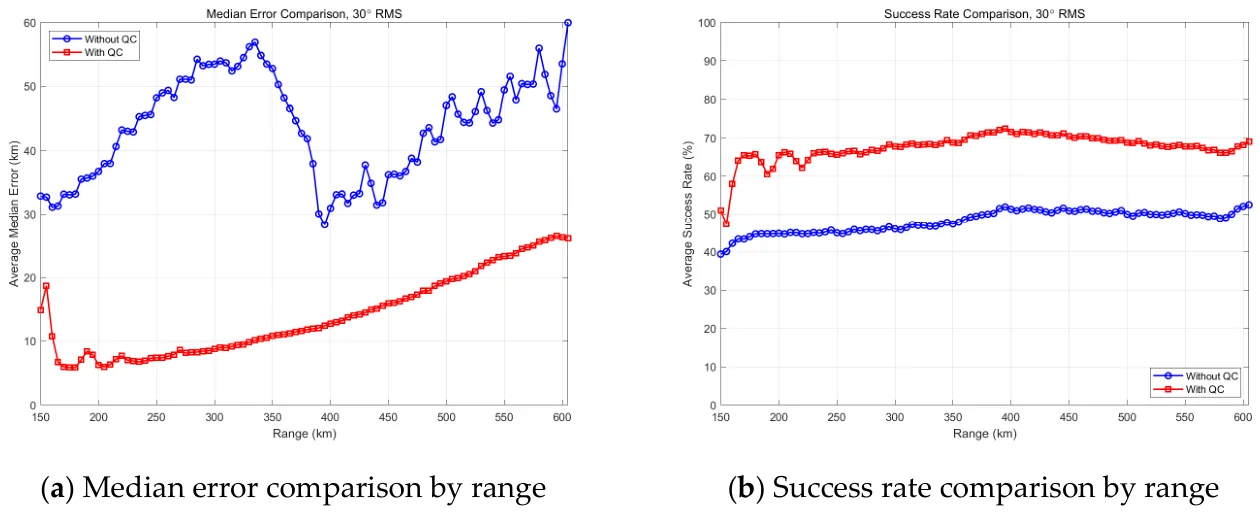
Comparison with and without quality control (single-difference phase noise of 30° RMS).

Figure 10 and Figure 11 compare the localization performance with and without quality control over the observation range of R ≥ 150 km. Figure 10a and Figure 10b show the median localization error and the success rate versus range under 10° RMS phase noise, respectively. Figure 11a,b show the corresponding median localization error and success rate versus range under 30° RMS phase noise. In all subplots, the horizontal axis is range (km). The vertical axis is the median localization error (km) in Figure 11a and the success rate (%) in Figure 11b. The blue curve denotes the result without quality control, and the red curve denotes the result with quality control. A localization is considered successful when the 3D positioning error is less than 8% of the true range.

The simulation results show that under 10° RMS phase noise, after applying quality control, the success rate increases from 87.04% to 94.07%, and the median localization error decreases from 6.30 km to 6.03 km. Under 30° RMS phase noise, with stdR quality control, the success rate increases from 48.10% to 68.11%, and the median localization error drops from 38.06 km to 13.02 km. For a clearer comparison, the key metrics are summarized in Table 4. The stdR quality control significantly enhances the credibility of the system’s positioning output.

### 3.4. Adaptability Analysis of Quality Control to TDOA Direction-Finding Error

To evaluate the adaptability of the proposed stdR quality control under the condition of increased azimuth measurement error, this subsection changes the TDOA direction-finding error to 3° RMS while keeping all other parameters identical to those in Section 3.1. The phase noise is set to 10° RMS, and the station location error is 1 mm RMS; the success criterion is 8% of the true range, and the quality-control threshold follows Equation (24). For this case, 200 Monte Carlo trials are performed over the same range–azimuth grid.

**Figure 12:**
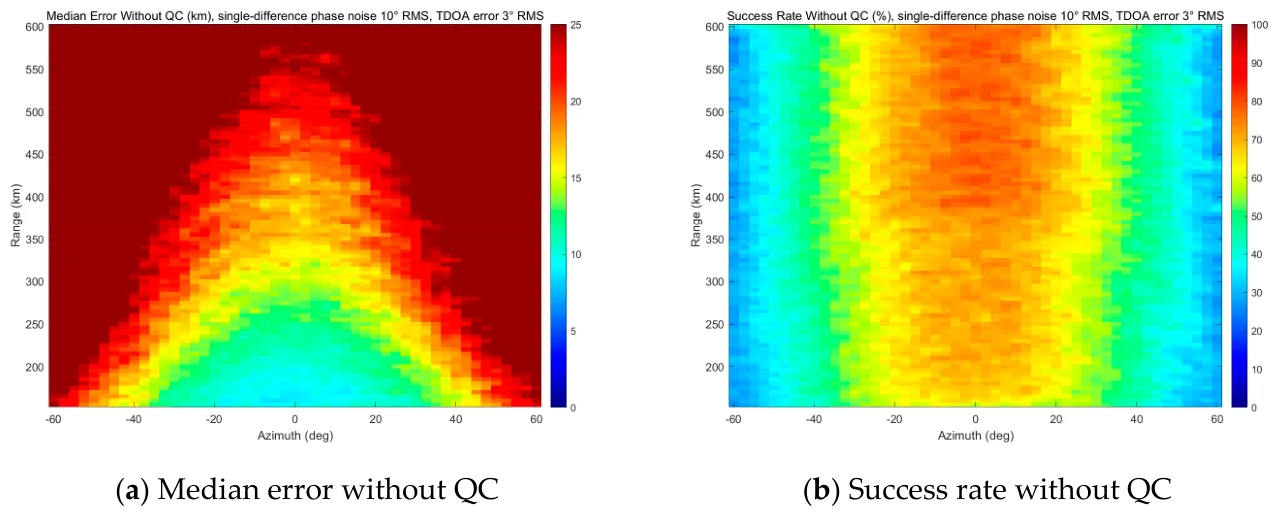
Localization error distribution and success rate without QC under 3° RMS TDOA direction-finding error (single-difference phase noise of 10° RMS).

Figure 12 presents the localization performance without quality control under a 3° RMS TDOA direction-finding error. Figure 12a shows the median localization error distribution, and Figure 12b shows the success rate. The single-difference phase noise is 10° RMS. A localization is considered successful if the localization error is within 8% of the true range. Only samples with R ≥ 150 km are counted.

**Figure 13:**
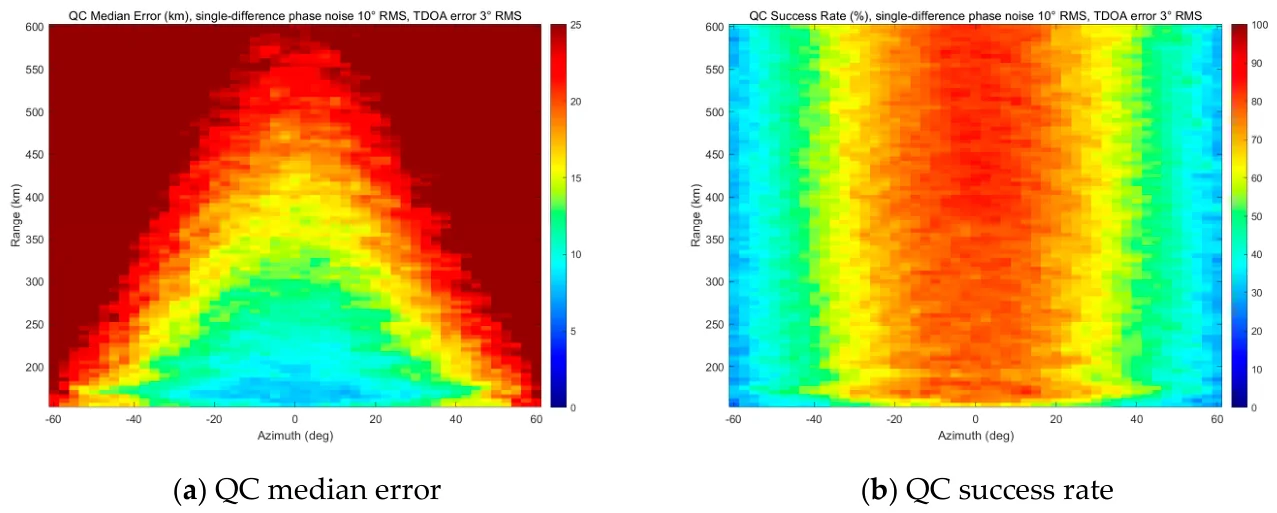
Localization error distribution and success rate with quality control under 3° RMS TDOA direction-finding error (single-difference phase noise of 10° RMS).

The stdR quality-control threshold follows Equation (24). Figure 13 presents the localization performance with quality control under the same condition. Figure 13a shows the median localization error distribution, and Figure 13b shows the success rate. Comparing Figure 13 with Figure 12, the median error is reduced, and the success rate is improved across the observation region.

**Figure 14:**
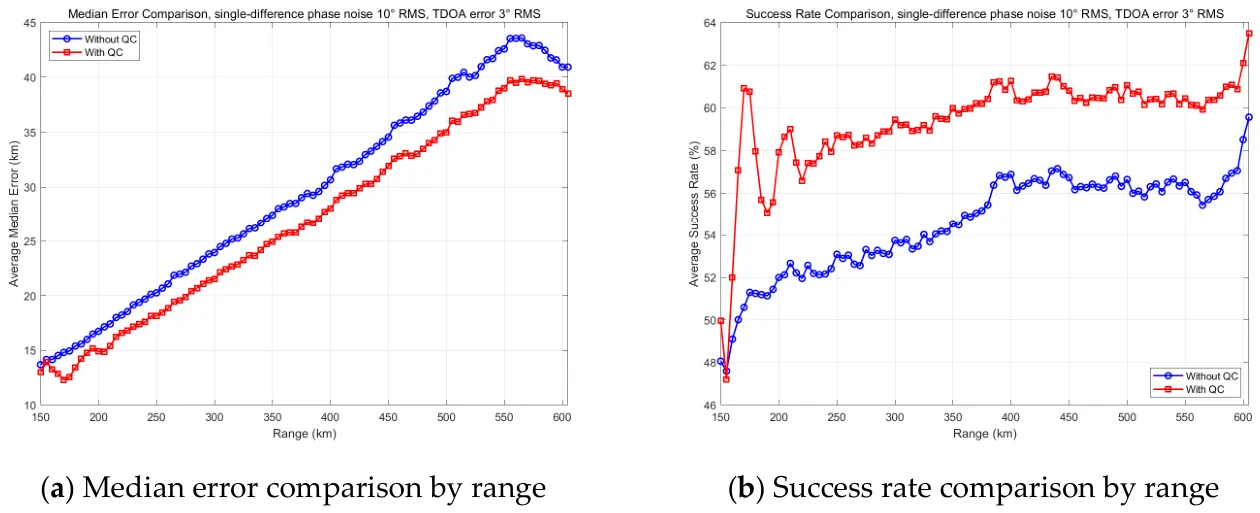
Comparison with and without QC under 3° RMS TDOA direction-finding error (single-difference phase noise of 10° RMS).

Figure 14 compares the localization performance with and without quality control over the observation range of R ≥ 150 km. Figure 14a shows the median localization error versus range, and Figure 14b shows the success rate versus range. The TDOA direction-finding error is 3° RMS, the single-difference phase noise is 10° RMS, and the success criterion is 8% of the true range.

The simulation results show that under an increased TDOA direction-finding error, specifically a 3° RMS TDOA direction-finding error, together with 10° RMS single-difference phase noise, after applying quality control, the success rate increases from 54.57% to 60.03%, and the median localization error decreases from 25.41 km to 23.34 km. For a clearer comparison, the key metrics are summarized in Table 5. The stdR quality control still improves the localization result when the TDOA direction-finding error is increased.

### 3.5. Comparison with the Cascade Ambiguity Resolution Method

To further evaluate the performance of the overall framework relative to existing approaches, this subsection compares the proposed progressive dPDOA method with a representative baseline, the Cascade Ambiguity Resolution method (referred to as “Cascade”), which is typically based on equal-length baselines [11]. The specific station coordinates and baseline combinations for the Cascade method are detailed in Table 6 and Table 7, respectively. All simulation parameters and error conditions remain identical to those in Section 3.1. Since the Cascade method fails to provide an output when the ambiguity resolution chain breaks, the statistical results for the Cascade method only evaluate the successfully resolved samples.

**Figure 15:**
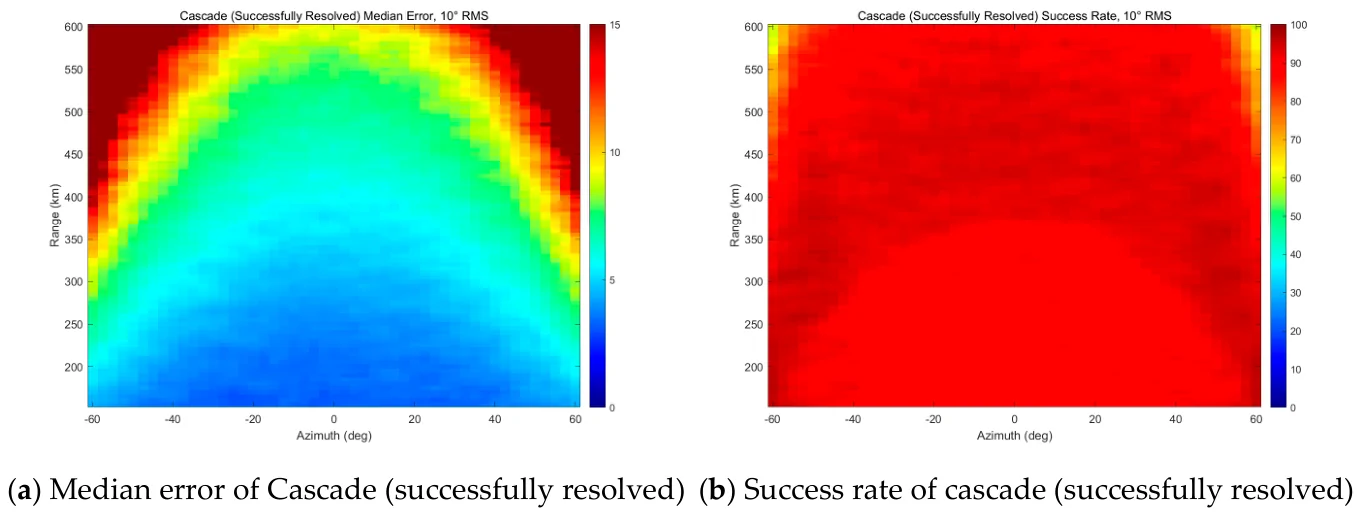
Localization performance of the Cascade method (single-difference phase noise of 10° RMS).

Figure 15 shows the localization performance of the Cascade method over the observation range of R ≥ 150 km under 10° RMS phase-difference measurement noise. Figure 15a and Figure 15b present the median localization error distribution and the success rate, respectively. It should be noted that the statistical results for the Cascade method are evaluated only on successfully resolved samples, excluding invalid samples where the ambiguity resolution chain failed to provide an output. In Figure 15a, the horizontal axis is azimuth (deg), the vertical axis is range (km), and the colorbar denotes the median localization error (km). In Figure 15b, the horizontal axis is azimuth (deg), the vertical axis is range (km), and the colorbar denotes the success rate (%). A localization is considered successful when the 3D positioning error is less than 8% of the true range.

**Figure 16:**
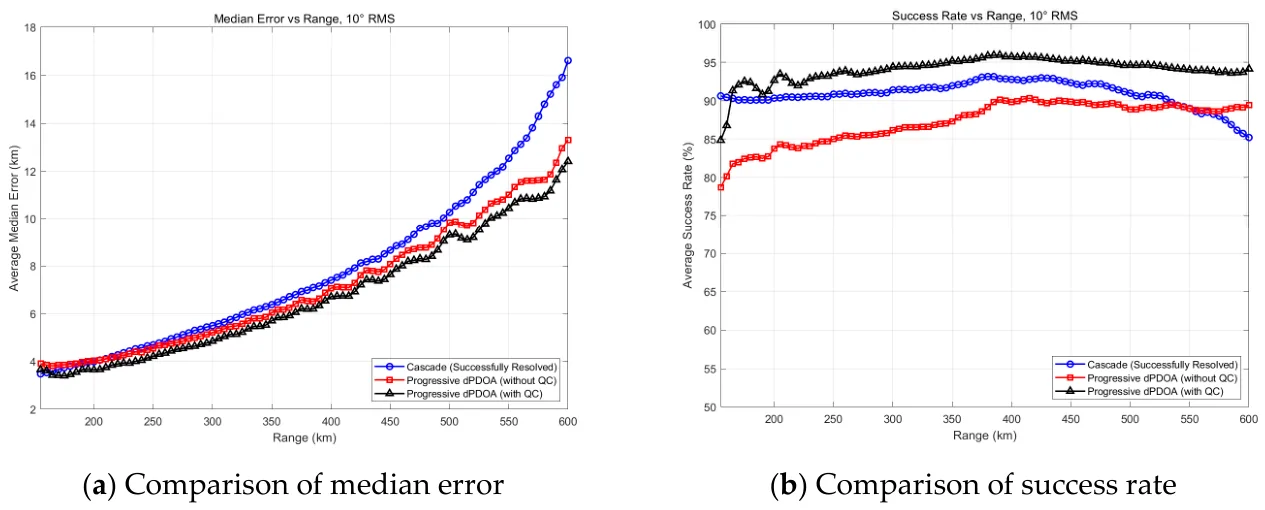
Comparison of localization performance between the Cascade method and Progressive dPDOA (single-difference phase noise of 10° RMS).

Figure 16 compares the localization performance of the Cascade method and the Progressive dPDOA method over the observation range of R ≥ 150 km under 10° RMS phase-difference measurement noise. Figure 16a and Figure 16b show the average median localization error and the average success rate versus range, respectively. In all subplots, the horizontal axis is range (km). The vertical axis is the average median error (km) in Figure 16a and the average success rate (%) in Figure 16b. The blue curve with circles denotes the Cascade method (evaluated only on successfully resolved samples), the red curve with squares denotes the Progressive dPDOA method without QC, and the black curve with triangles denotes the Progressive dPDOA method with QC.

**Figure 17:**
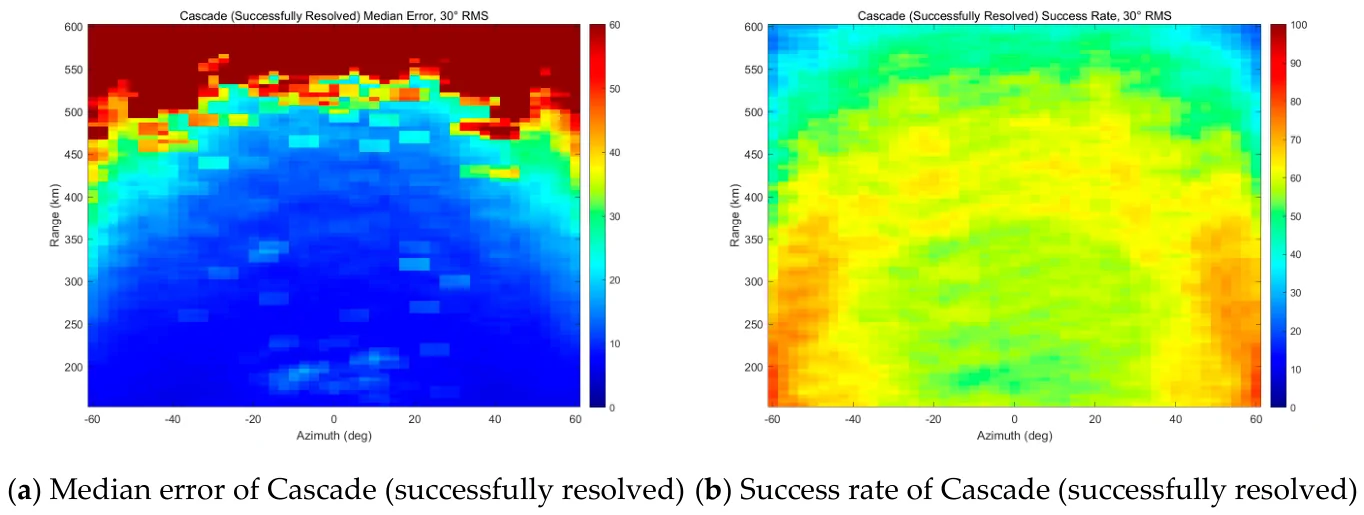
Localization performance of the Cascade method (single-difference phase noise of 30° RMS).

**Figure 18:**
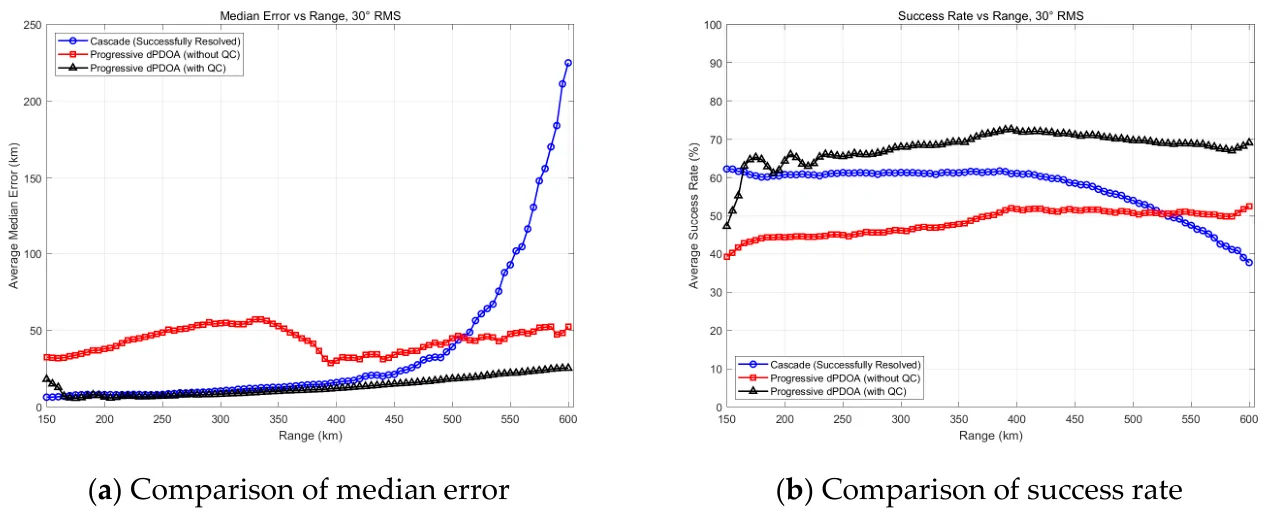
Comparison of localization performance between the Cascade method and Progressive dPDOA (single-difference phase noise of 30° RMS).

Figure 17 presents the localization performance distribution of the Cascade method under 30° RMS phase noise. Figure 17a and Figure 17b present the median localization error distribution and the success rate, respectively. Figure 18 compares the localization performance of the Cascade method and the Progressive dPDOA method under 30° RMS phase noise. Figure 18a and Figure 18b show the average median localization error and the average success rate versus range, respectively. Compared with Figure 15, the high-precision region in Figure 17a shrinks significantly under 30° RMS noise, with the median error at the peripheral regions exceeding 60 km. Figure 17b indicates that the success rate in the central area drops to 50–70%. Similarly, in Figure 18a, the Cascade curve shows a sharp divergence beyond 400 km, while the Progressive dPDOA with QC maintains a stable error below 20 km across the entire range.

Table 8 summarizes the overall performance comparison among the Cascade method, Progressive dPDOA without QC, and Progressive dPDOA with QC under both 10° and 30° RMS phase noise.

Under the 10° RMS phase noise condition, the Progressive dPDOA method with QC achieves a retained-sample success rate of 94.07%, which is 2.99 percentage points higher than the Cascade method (91.08%). Similarly, its overall success rate reaches 71.07%, outperforming the Cascade method (68.20%) by 2.87 percentage points. However, its retained-sample median error (6.03 km) is marginally higher by 0.13 km.

Under the extreme 30° RMS phase noise condition, the Progressive dPDOA method with QC demonstrates a more significant advantage. Its retained-sample success rate reaches 68.11%, which is 9.94 percentage points higher than that of the Cascade method (58.17%). Furthermore, its retained-sample median error is 13.02 km, which is 1.48 km lower than that of the Cascade method (14.50 km), indicating higher accuracy. Although its overall success rate (28.87%) is 6.71 percentage points lower than that of the Cascade method (35.58%), this reduction is a direct consequence of deliberately rejecting unreliable samples rather than a degradation of localization capability. The objective of this work is not to maximize the overall success rate over all trials but to increase the retained-sample success rate so as to provide more accurate and reliable localization points for subsequent track filtering. Since a radar typically emits hundreds to thousands of pulses per second, each of which can produce one localization point, the number of available localization points is sufficiently large; in this context, retaining a smaller set of high-confidence points is more valuable than retaining a larger set mixed with unreliable ones. This also explains the fundamental difference between the two methods: the Cascade method frequently collapses and fails to output when its ambiguity chain breaks, whereas the Progressive dPDOA method with QC actively rejects unreliable samples to guarantee the credibility of its retained outputs.

Although the global median error in Table 8 is 14.50 km, it is worth noting that the spatial average error across ranges is 32.64 km. As depicted in Figure 18a, the error at 600 km diverges sharply to over 200 km, highlighting the fragility of the cascade chain at long ranges.

As illustrated in Figure 18b, under the 30° RMS phase noise condition, the advantage of the Progressive dPDOA method with QC in terms of the retained-sample success rate becomes increasingly prominent as the range increases. At 600 km, the retained-sample success rate of the Progressive dPDOA method with QC remains at approximately 69%, which is about 31 percentage points higher than that of the Cascade method (which drops to approximately 38%).

### 3.6. Computational Complexity Analysis

The computational cost of the proposed method is analyzed as follows. Let *K* be the number of height hypotheses, *N_R_* the number of range grid points, *N_A_* the number of azimuth grid points in a conventional three-dimensional grid search, *S* the number of dPDOA observations, *M* the number of retained baselines, *L* the ambiguity dimension, *I* the number of nonlinear least-squares iterations, and *N* the number of signal samples.

The TDOA coarse direction finding computes the cross-correlation of the longest baseline; when implemented by FFT, its computational complexity is *O*(*N*log*N*). The multi-height coarse search performs, for each of the *K* height hypotheses, a one-dimensional range search over *N_R_* grid points and evaluates the cost function over *S* dPDOA observations, so its complexity is *O*(*KN_R_*S). Since the azimuth is fixed by the TDOA prior, no azimuth grid search is required, and the azimuth dimension is effectively removed. The stdR quality control reuses the coarse-search results, and its additional computation is *O*(1). The LAMBDA ambiguity fixing performs an integer least-squares search in the ambiguity domain; after integer decorrelation and tree search, its complexity is typically *O*(*L*^3^). The nonlinear least-squares refinement requires *I* iterations, each involving the evaluation of *M* residuals and the corresponding Jacobian, so its complexity is *O*(*IM*).

Therefore, the overall computational complexity of the proposed method is approximately *O*(*N*log*N* + *KN_R_*S + *L*^3^ + *IM*), in which the dominant terms are *KN_R_*S and *L*^3^, while the remaining terms are of lower order. For comparison, a direct three-dimensional grid search over range, azimuth, and height would require *O*(*KN_R_N_A_*S). Since *N_A_* is usually much larger than 1, the proposed progressive chain significantly reduces the search space by compressing the 3D search into a set of one-dimensional range searches along the TDOA azimuth ray. Moreover, the stdR quality control requires no extra computation because it fully reuses the intermediate results of the coarse search. Consequently, the proposed method improves localization reliability without increasing the computational burden, which is favorable for practical applications.

## 4. Discussion

In the progressive localization chain, quality control is a key step that determines the reliability of the final output. To this end, a pre-quality-control metric based on the standard deviation of range estimates across heights (stdR) is proposed. This metric uses the consistency of range estimates under several typical height hypotheses to judge the reliability of the localization result: a real target should give similar range estimates under different heights, while a false minimum may appear only under a specific height hypothesis, causing the estimated ranges to diverge. The stdR metric reuses the intermediate results of the coarse search and adds negligible computation. Quantitatively, under 10° RMS single-difference phase noise, the stdR quality control increases the success rate from 87.04% to 94.07% and reduces the median localization error from 6.30 km to 6.03 km. Under 30° RMS single-difference phase noise, it increases the success rate from 48.10% to 68.11% and reduces the median localization error from 38.06 km to 13.02 km. Under a 3° RMS TDOA direction-finding error and 10° RMS phase noise, it increases the success rate from 54.57% to 60.03% and reduces the median localization error from 25.41 km to 23.34 km. The above quantitative results show that the stdR quality control effectively improves the success rate and reduces the median localization error.

A comparison between Section 3.3 and Section 3.4 demonstrates that accurate TDOA measurement is a critical stage of the progressive chain. Under 10° RMS single-difference phase noise, when the TDOA direction-finding error RMS increases from 0.143° to 3°, the success rate with stdR QC decreases sharply from 94.07% to 60.03%, while the median error increases from 6.03 km to 23.34 km. This confirms that TDOA direction finding is an indispensable step and a key stage that constrains the search space and supports the subsequent coarse-to-fine localization. Section 3.3 further quantifies the difference with and without stdR QC, showing that stdR significantly improves reliability under both 10° and 30° RMS phase noise. The proposed method should therefore be regarded as an integrated progressive framework rather than a mere serial combination of existing techniques. Each stage has a clear input–output relationship and a distinct role: TDOA direction finding provides the azimuth prior and compresses the 3D search into a ray; the multi-height coarse search handles the unknown target height and provides the range-consistency statistics for stdR; stdR rejects false-peak samples before ambiguity fixing; LAMBDA resolves the integer ambiguities; and nonlinear least-squares refinement yields the final high-precision position. Omission or failure of any stage can lead to sharp performance degradation.

Furthermore, the comparison with the traditional Cascade Ambiguity Resolution method highlights the fundamental advantage of the proposed progressive architecture. The Cascade method relies on hierarchical short-to-long baseline resolution, which is highly sensitive to phase noise and degrades catastrophically at long ranges due to phase sensitivity attenuation. As demonstrated in the simulations, under 30° RMS phase noise, the overall success rate of the Cascade method plummets to 35.58%, and its error diverges sharply beyond 400 km. In contrast, the progressive dPDOA framework fundamentally overcomes this bottleneck. By integrating TDOA coarse direction finding, multi-height search, and LAMBDA global ambiguity fixing, it not only maintains a high retained-sample success rate (68.11% with QC, outperforming Cascade by 9.94 percentage points) but also ensures a reliable absolute output rate. Although the overall success rate of the Progressive dPDOA method with QC (28.87%) is slightly lower than Cascade’s (35.58%), their failure mechanisms are fundamentally different: Cascade fails due to resolution chain collapse, whereas the proposed method actively rejects unreliable samples through the stdR threshold to guarantee the credibility of its outputs.

However, the proposed method also has clear limitations. The progressive localization method relies on a TDOA azimuth prior. When the TDOA direction-finding error is large, the search space is no longer tightly constrained, and the localization performance degrades. Under extremely large phase noise, false peaks may dominate the cost function, and the stdR metric may be insufficient to reject all unreliable samples. These limitations define the scope of the proposed method and point to future work, including multi-frequency assistance, kinematic constraints, and robust ambiguity resolution.

## 5. Conclusions

Based on the above discussion, this paper develops a progressive fusion framework for dPDOA localization. The framework uses TDOA direction finding to obtain an azimuth prior, compresses the 3D search into a ray, obtains a reliable coarse position through one-dimensional range searches under multiple height hypotheses, provides a high-quality initial point for LAMBDA ambiguity fixing, and finally refines the position by nonlinear optimization. Each step has a clear input–output relationship, which facilitates convergence and provides intervention points for quality control. Comparative simulations with the conventional Cascade Ambiguity Resolution method demonstrate that the proposed progressive framework effectively overcomes the fragility of traditional hierarchical ambiguity resolution under long-range and heavy-noise conditions. The core novelty of this work is the proposed stdR-based quality-control metric, which rejects false samples caused by cost-function multimodality before the complex ambiguity-fixing stage, thereby ensuring high output reliability and significantly improving the overall positioning performance.

## 6. Future Work

Future work will focus on two aspects. To address the false-peak problem under large phase-difference measurement errors, a post-processing residual test after nonlinear least-squares refinement can be added to complement the stdR pre-quality control. This will construct a reliability assurance mechanism from two dimensions: geometric consistency of positioning and matching degree between residuals and theoretical noise statistics [24], enabling layer-by-layer identification and rejection of failed samples. In addition, robust ambiguity resolution methods based on integer range constraints, multi-frequency assistance, or kinematic constraints will be investigated to improve the positioning success rate under extreme conditions. Specific directions include the following: exploiting multi-frequency characteristics to form additional constraint equations and increase ambiguity resolution redundancy [25]; introducing target kinematic models to achieve recursive ambiguity fixing and validation within a Kalman filter framework; and exploring search-space pruning strategies in integer least squares to reduce the ambiguity candidate set size and the risk of incorrect fixing [21].

## Figures and Tables

**Table 1:** Station coordinates.

Station ID	X (m)	Y (m)	Z (m)	Description
1	0.000	0.000	0.000	Center station
2	0.150	0.000	0.000	Horizontal slave (0.15 m)
3	−0.150	0.000	0.000	Horizontal slave (0.15 m)
4	2.000	0.000	0.000	Horizontal slave (2 m)
5	−2.000	0.000	0.000	Horizontal slave (2 m)
6	5.000	0.000	0.000	Horizontal slave (5 m)
7	−5.000	0.000	0.000	Horizontal slave (5 m)
8	11.000	0.000	0.000	Horizontal slave (11 m)
9	−11.000	0.000	0.000	Horizontal slave (11 m)
10	37.000	0.000	0.000	Horizontal slave (37 m)
11	−37.000	0.000	0.000	Horizontal slave (37 m)
12	83.000	0.000	0.000	Horizontal slave (83 m)
13	−83.000	0.000	0.000	Horizontal slave (83 m)
14	181.000	0.000	0.000	Horizontal slave (181 m)
15	−181.000	0.000	0.000	Horizontal slave (181 m)
16	307.000	0.000	0.000	Horizontal slave (307 m)
17	−307.000	0.000	0.000	Horizontal slave (307 m)
18	0.000	0.000	50.000	Elevated station

Note: The array is a linear configuration along the *x*-axis. The elevated station provides vertical sensitivity through a 50 m height offset.

**Table 2:** Baseline pairs (dPDOA groups).

Group ID	Center Station	Slave Station 1	Slave Station 2	Type
1	1	2	3	Horizontal (0.15 m)
2	1	4	5	Horizontal (2 m)
3	1	6	7	Horizontal (5 m)
4	1	8	9	Horizontal (11 m)
5	1	10	11	Horizontal (37 m)
6	1	12	13	Horizontal (83 m)
7	1	14	15	Horizontal (181 m)
8	1	16	17	Horizontal (307 m)
9	18	2	3	Mixed (elevated + 0.15 m)
10	18	4	5	Mixed (elevated + 2 m)
11	18	6	7	Mixed (elevated + 5 m)
12	18	8	9	Mixed (elevated + 11 m)
13	18	10	11	Mixed (elevated + 37 m)
14	18	12	13	Mixed (elevated + 83 m)
15	18	14	15	Mixed (elevated + 181 m)
16	18	16	17	Mixed (elevated + 307 m)

Note: Each group consists of three stations forming a symmetric double-difference observation. Mixed groups take the elevated station (18) as the symmetry center, with two horizontal slave stations symmetric about it. The vertical sensitivity arises from the 50 m height difference between the elevated center and the horizontal slaves. The array targets a minimum observation range of 150 km. Near-range target ambiguity resolution requires more groups of short-range baselines. Since all horizontal groups share center station 1, and all mixed groups share center station 18, the combined noise terms of different dPDOA observations are correlated. This correlation is accounted for by Q*_ϵ_* in Equation (13).

**Table 3:** Simulation parameter settings.

Parameter	Value	Remarks
Signal wavelength, *λ*	0.0333 m	Corresponding to 9 GHz
Single-difference phase noise RMS	10° and 30°	Receiver noise
Site error RMS	1 mm	Residual after calibration
Channel phase deviation RMS	1.5°	Hardware and cable differences
TDOA direction-finding error RMS	0.143°	600 m baseline, 5 ns RMS time-difference error
Success threshold	8% of range	Relative positioning error for track initiation
Observation range	≥150 km	Minimum target range

**Table 4:** Overall performance with and without QC under different phase noise levels.

Noise (RMS)	Metric	Without QC	with QC	Improvement
10°	Success rate (%)	87.04	94.07	+7.03 pp
10°	Median error (km)	6.30	6.03	−0.27 km
10°	Retention rate (%)	—	81.65	—
30°	Success rate (%)	48.10	68.11	+20.01 pp
30°	Median error (km)	38.06	13.02	−25.04 km
30°	Retention rate (%)	—	60.03	—

Note: A localization is considered successful when the 3D positioning error is less than 8% of the true range. Only samples with R ≥ 150 km are considered. “pp” denotes percentage points. For the median error, negative values in the Improvement column indicate a reduction in localization error.

**Table 5:** Overall performance with and without QC under 3° RMS TDOA error.

TDOA Error (RMS)	Phase Noise (RMS)	Metric	Without QC	with QC	Improvement
3°	10°	Success rate (%)	54.57	60.03	+5.46 pp
3°	10°	Median error (km)	25.41	23.34	−2.07 km
3°	10°	Retention rate (%)	—	80.68	—

Note: A localization is considered successful when the 3D positioning error is less than 8% of the true range. Only samples with R ≥ 150 km are considered. “pp” denotes percentage points. For the median error, negative values in the Improvement column indicate a reduction in localization error.

**Table 6:** Station coordinates for the Cascade method.

Station ID	X (m)	Station ID	X (m)	Station ID	X (m)	Station ID	X (m)
1	0.000	6	−250.00	11	200.00	16	−99.38
2	−307.00	7	250.00	12	−198.14	17	99.38
3	307.00	8	−248.14	13	198.14	18	−25.00
4	−301.04	9	248.14	14	−100.00	19	25.00
5	301.04	10	−200.00	15	100.00		

Note: The array is a linear configuration along the *x*-axis. All stations have y = 0 and z = 0.

**Table 7:** Baseline pairs (Cascade method).

Group ID	Station A	Station B	Station C	Station D	Equivalent Half-Baseline (m)
1	2	4	5	3	Four-element virtual (da ≈60.2 m)
2	6	8	9	7	Four-element virtual (da ≈30.4 m)
3	10	12	13	11	Four-element virtual (da ≈27.2 m)
4	14	16	17	15	Four-element virtual (da ≈11.1 m)
5	1	2	3	—	Three-element baseline (307 m)
6	1	6	7	—	Three-element baseline (250 m)
7	1	10	11	—	Three-element baseline (200 m)
8	1	14	15	—	Three-element baseline (100 m)
9	1	18	19	—	Three-element baseline (25 m)

Note: The Cascade method utilizes a pure planar array configuration. Groups 1–4 are four-element virtual baseline groups used for ambiguity resolution. For these groups, Stations A and D form the outer symmetric pair, and Stations B and C form the inner symmetric pair. The effective baseline is defined as $d_{a}=\sqrt{d_{\mathrm{AD}}^{2}-d_{\mathrm{BC}}^{2}}$, and the corresponding geometric observation function is *R_A_* + *R_D_* − *R_C_* − *R_B_*. Groups 5–9 are three-element equal-length baselines used for fine ranging, where Station A is the center station, and Stations B and C are the symmetric slave stations. The corresponding geometric observation function for these groups is *R_B_* + *R_C_* − 2*R_A_*.

**Table 8:** Overall performance comparison among Cascade, Progressive dPDOA without QC, and Progressive dPDOA with QC.

Noise (RMS)	Metric	Cascade (Valid Samples)	Progressive dPDOA (Without QC)	Progressive dPDOA (with QC)
10°	Retained-sample success rate (%)	91.08	87.04	94.07
10°	Retained-sample median error (km)	5.90	6.30	6.03
10°	Overall success rate (%)	68.20	87.04	71.07
30°	Retained-sample success rate (%)	58.17	48.10	68.11
30°	Retained-sample median error (km)	14.50	38.06	13.02
30°	Overall success rate (%)	35.58	48.10	28.87

Note: The table reports three metrics: the retained-sample success rate, the retained-sample median error, and the overall success rate (calculated against all Monte Carlo trials). The retained-sample median error is calculated globally across all valid samples. Due to the large number of valid samples in the near-to-mid range (150–450 km, accounting for over 75%), the global median for the Cascade method at 30° RMS is significantly suppressed to 14.50 km, masking its actual divergence at long ranges.

## Data Availability

The data presented in this study are available on request from the corresponding author due to institutional data protection policies.

## Abbreviations

The following abbreviations are used in this manuscript:

PDOA	Phase Difference of Arrival
dPDOA	Differential Phase Difference of Arrival
TDOA	Time Difference of Arrival
LAMBDA	Least-Squares Ambiguity Decorrelation Adjustment
stdR	Standard Deviation of Range Estimates Across Heights
RMS	Root Mean Square
UAV	Unmanned Aerial Vehicle
FDOA	Frequency Difference of Arrival
CRLB	Cramér–Rao Lower Bound
SNR	Signal-to-Noise Ratio
PDOP	Position Dilution of Precision
